# A 37-amino acid loop in the *Yarrowia lipolytica* hexokinase impacts its activity and affinity and modulates gene expression

**DOI:** 10.1038/s41598-021-85837-8

**Published:** 2021-03-19

**Authors:** Piotr Hapeta, Patrycja Szczepańska, Cécile Neuvéglise, Zbigniew Lazar

**Affiliations:** 1 Department of Biotechnology and Food Microbiology, Faculty of Biotechnology and Food Science, Wrocław University of Environmental and Life Sciences, Chełmońskiego 37, 51-630 Wrocław, Poland; 2 SPO, INRAE, Montpellier SupAgro, Univ Montpellier, 34060 Montpellier, France

**Keywords:** Biochemistry, Biotechnology, Cell biology, Computational biology and bioinformatics, Genetics, Microbiology, Molecular biology

## Abstract

The oleaginous yeast *Yarrowia lipolytica* is a potent cell factory as it is able to use a wide variety of carbon sources to convert waste materials into value-added products. Nonetheless, there are still gaps in our understanding of its central carbon metabolism. Here we present an in-depth study of *Y. lipolytica* hexokinase (YlHxk1), a structurally unique protein. The greatest peculiarity of YlHxk1 is a 37-amino acid loop region, a structure not found in any other known hexokinases. By combining bioinformatic and experimental methods we showed that the loop in YlHxk1 is essential for activity of this protein and through that on growth of *Y. lipolytica* on glucose and fructose. We further proved that the loop in YlHxk1 hinders binding with trehalose 6-phosphate (T6P), a glycolysis inhibitor, as hexokinase with partial deletion of this region is 4.7-fold less sensitive to this molecule. We also found that YlHxk1 devoid of the loop causes strong repressive effect on lipase-encoding genes *LIP2* and *LIP8* and that the hexokinase overexpression in *Y. lipolytica* changes glycerol over glucose preference when cultivated in media containing both substrates.

## Introduction

*Yarrowia lipolytica* is an oleaginous yeast that has become a biotechnological workhorse due to its industrially-relevant abilities. This yeast synthesize high concentration of intracellular lipids and secrete high amount of proteins as well as organic acids and polyols^1–6^. *Y. lipolytica* is able to use both hydrophobic (e.g.* n-*alkanes, lipids) and hydrophilic (e.g. monosaccharides, glycerol, organic acids) carbon sources^7–12^. However, wild strains of *Y. lipolytica* utilize only a handful of sugar substrates, namely glucose, fructose and mannose. These hexoses are transported inside the cell via hexose transporters and incorporated to the central carbon metabolism after their phosphorylation by hexose kinases. This yeast has two enzymes with hexokinase activity—glucokinase (YlGlk1) and hexokinase (YlHxk1). YlGlk1 exclusively catalyses the phosphorylation of glucose, while YlHxk1 acts on glucose and fructose^13^. This is in contrast with the model yeast *Saccharomyces cerevisiae*, which possesses two hexokinases—ScHxk1 and ScHxk2, which derive from the whole genome duplication (WGD^14^). ScHxk2 plays the main role in glucose phosphorylation and participates in glucose catabolic repression in *S. cerevisiae*^15,16^.

YlHxk1 is a 534 amino acid protein encoded by a 2041 bp *YlHXK1* gene (*YALI0B22308g*) containing an intron^13^. The deletion of *YlHXK1* extends the doubling time of the corresponding mutant by approximately 15% compared to the wild strain and makes the yeast unable to grow on fructose^13^. *Y. lipolytica* hexokinase has a Km of 0.38 mM and 3.56 mM for glucose and fructose, respectively^13^. YlHxk1 is extremely sensitive to trehalose-6-phoshpate (T6P), a yeast glycolytic inhibitor, exhibiting a Ki of 0.0036 mM^13,17^. To date, no other studied hexokinase showed this level of inhibition. Overexpression of *YlHXK1* substantially increases carbon flux through glycolysis^18^, enhances sugar utilization, reduces filamentation, and improves lipid accumulation from fructose in a high-lipid accumulating strain up to 55%^19^. Additionally, YlHxk1 successfully substitutes hexokinase II from *S. cerevisiae* (ScHxk2) in glucose catabolite repression of invertase (encoded by *SUC2* gene), what indicates the bifunctionality of this protein^13^. Furthermore, Fickers and colleagues^20^ reported that the expression of *LIP2* gene, encoding an extracellular lipase in *Y. lipolytica*, is repressed by glucose and that YlHxk1 is involved in this process. It was also reported that YlHxk1 affects expression of several genes involved in erythritol biosynthesis and tricarboxylic acid cycle^9^. This observation, together with four potential nuclear localization sequences (NLS) in the primary structure of YlHxk1^13^ indicates a bifunctional role of *Y. lipolytica* hexokinase similar to that of ScHxk2. Moreover, these yeasts show a peculiar carbon source utilization pattern, i.e. it utilizes glycerol first followed by glucose consumption^21^. Recent findings suggest that YlHxk1 might be involved in the regulation of this process^9^.

As in many known hexokinases, including ScHxk1, ScHxk2 and *Kluyveromyces lactis* hexokinase (KlHxk1), the YlHxk1 protein has conserved sugar and ATP binding sequences^13,22,23^. In ScHxk2, there are amino acid residues crucial for its catalytic activity. For example, D343 (D386 in YlHxk1) is a residue in the heart of the large lobe, away from the sugar binding cleft while E457 (E500 in YlHxk1) is a part of the highly conserved motif in hexokinase family proteins ^457^EDGSGAGAAV^466^ (^500^EDGSGVGAAL^509^ in YlHxk1) at the C-terminal end^24^. Mutations of these residues severely affect catalytic activity and substrate recognition and were reported to change the derepression of *SUC2* by glucose as in *S. cerevisiae hxk2* null mutant^24^. On the other hand, the S15 is a phosphorylation/dephosphorylation site required for shuttling back and forth of the protein between the nucleus and the cytoplasm^25^ while the amino acids 7–15 function as NLS in ScHxk2^26^. Although these residues are conserved in YlHxk1, no reports on their functions were provided so far.

The most interesting feature of YlHxk1 is a sequence of 37 amino acids forming a loop structure not present in other known and characterized hexokinases^13^. The loop region is located after the 146^th^ amino acid residue and flanked by the ATP and glucose binding domains. Until now, the function of this element remains elusive.

Here, we report the structure–function analysis of the hexokinase YlHxk1 of *Y. lipolytica*. The main emphasis is put on the characterization of the 37-amino acid loop and determination of its potential functions. Both bioinformatic and experimental data are provided to deepen our knowledge on the influence of hexokinase on the central carbon metabolism in *Y. lipolytica*. Additionally, the kinetic parameters of hexokinase from *Yarrowia yakushimensis,* the only protein among the *Yarrowia* clade missing the mysterious loop region, are provided.

## Results

### The loop region is present in most of the *Yarrowia* clade yeast species hexokinases

**Figure 1:**
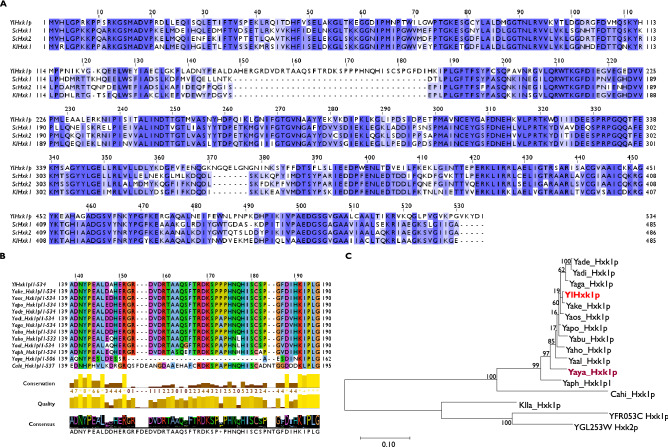
(**A**) Sequence alignment of YlHxk1, ScHxk1, ScHxk2 and KlHxk1; (**B**) sequence alignment of the loop region of hexokinases in the *Yarrowia* clade; (**C**) phylogenetic tree of the hexokinases based on Maximum likelihood. Bootstrap values are shown next to the branches. The tree is drawn to scale, with branch lengths representing the number of substitutions per site.

**Figure 2:**
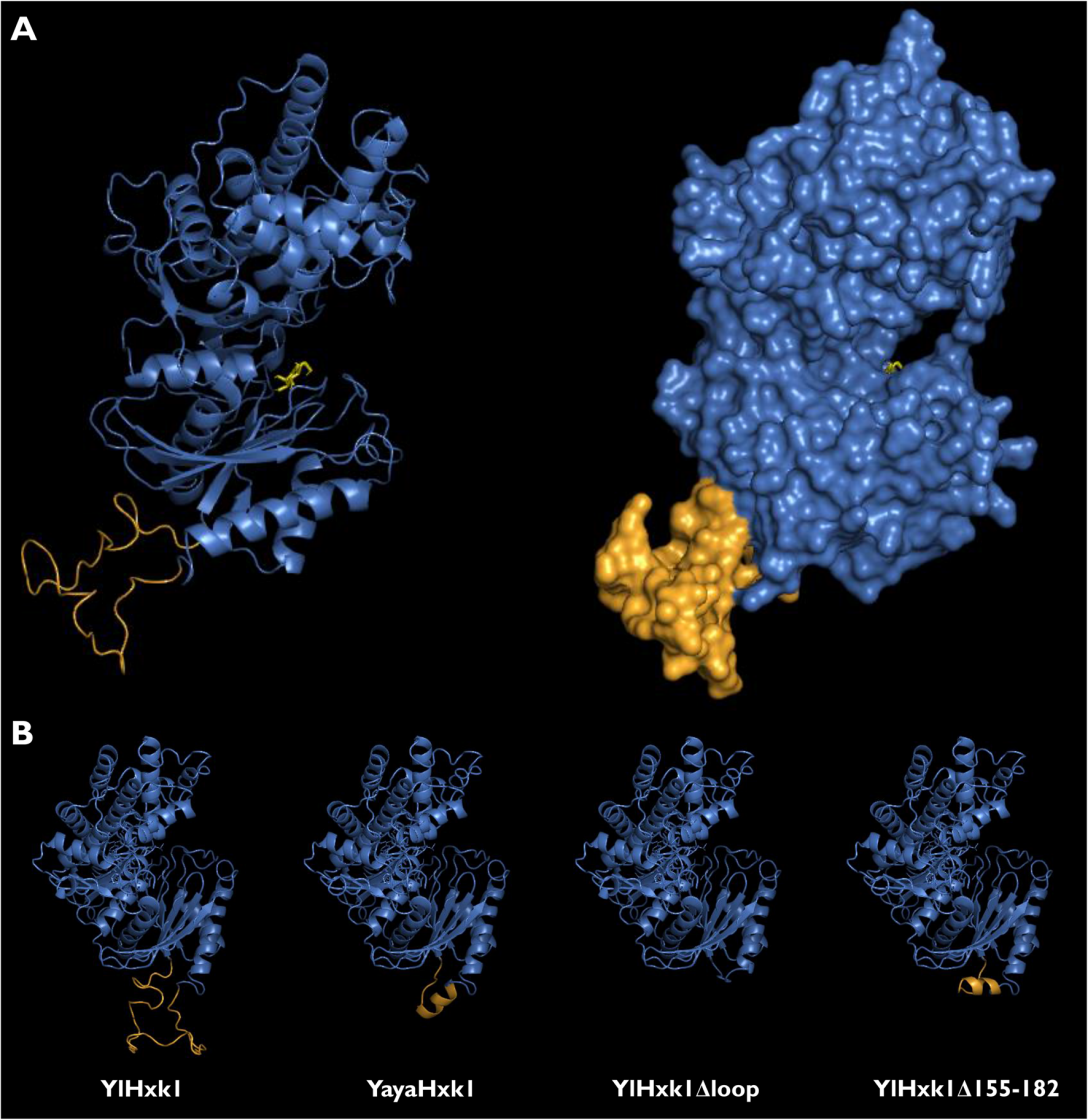
(**A**) Three-dimensional models of YlHxk1 (main structure in blue and the loop in orange) in complex with glucose (yellow); (**B**) comparison of YlHxk1, YayaHxk1 and modified YlHxk1 structures.

The BLAST analysis of YlHxk1, ScHxk1, ScHxk2 and KlHxk1 showed that the additional amino acid region in YlHxk1 missing in other hexokinases includes 37 residues (^148^A–^184^I; Fig. 1A). Here, we were interested whether this unusual element constitutes a general rule in hexokinases of the yeast belonging to the *Yarrowia* clade^27^. Sequences alignment (Fig. 1B) of YlHxk1p with 12 other hexokinases showed that the loop is present in all analysed sequences but YayaHxk1p, a *Y. yahushimensis* hexokinase, in which the loop region is composed of 9 instead of 37 amino acids. The phylogenetic tree (Fig. 1C) and the distance matrix (Supplementary Table S1) showed that YayaHxk1p along with YaphHxk1p and CahiHxk1p are the most distant proteins to YlHxk1. Using SWISS-MODEL (https://swissmodel.expasy.org/) a three-dimensional YlHxk1p model has been built on a *K. lactis* KlHxk1p hexokinase template (Fig. 2A). The predicted structure shows that the loop is conformationally located outside the catalytic domains and seemingly takes no part in catalytic functions of the enzyme.

### The loop in YlHxk1 is indispensable for growth on glucose and fructose

To characterize YlHxk1 and to infer biological functions of its elements, a set of *Y. lipolytica* transformants expressing native and modified YlHxk1 as well as heterologous hexokinases from *S. cerevisiae* (ScHxk2) and *Y. yakushimensis* (YayaHxk1) under the control of constitutive TEF promoter (to avoid promoter impact on gene expression) were constructed in a *Y. lipolytica* Po1d *hxk1 glk1* genetic background (Table 1). Constructed strains can be divided into three groups: (1) expressing native hexokinases (YlHxk1, ScHxk2, YayaHxk1); (2) expressing YlHxk1 with deletion of large amino acid sequences (YlHxk1Δ7-15, YlHxk1Δloop, YlHxk1Δ155-182); and (3) expressing YlHxk1 with point mutations (YlHxk1 S15A, YlHxk1 D386E, YlHxk1 E500G, YlHxk1 D386E E500G). Due to the randomness of overexpression cassette incorporation into the genome, three strains of each gene variant were created, resulting in a total of 30 *Y. lipolytica* strains. Apart from YlHxk1Δloop and YlHxk1Δ155-182, which were created to infer the loop’s role(s), the mutations were introduced in order to compare functions of particular amino acids and sequences to the well-known ScHxk2.

**Table 1:** Strains used in this study.

Strain	Genotype	Antibiotic resistance/auxotrophy	Source or reference
***E. coli strains***			
DH5α	*fhuA2 lac(del)U169 phoA glnV44 Φ80′ lacZ(del)M15 gyrA96 recA1 relA1 endA1 thi-1 hsdR17*	–	Thermo Fisher Scientific
E2101	DH5α JMP62-URA3-pTEF	kanR	^50^
E2901	DH5α pCR™-Blunt II TOPO™ PUT-*GLK1*		This work
ZLE15	DH5α pCR™-Blunt II TOPO™ PUT-*GLK1*		
ZLE13	DH5α JMP62URA3ex-pTEF-Sc*HXK2*		This work
ZLE2103	DH5α JMP62URA3-pTEF-Yl*HXK1*		
ZLE2105	DH5α JMP62URA3ex-pTEF-Yl*HXK1*-S15A		
ZLE2107	DH5α JMP62URA3ex-pTEF-Yl*HXK1*Δ7-15		
ZLE2308	DH5α JMP62URA3ex-pTEF-Yl*HXK1*-Δloop		
ZLE2309	DH5α JMP62URA3ex-pTEF-Yl*HXK1-*Δ155-182		
ZLE2310	DH5α JMP62URA3ex-pTEF-Yl*HXK1*-D386E		
ZLE2311	DH5α JMP62URA3ex-pTEF-Yl*HXK1*-E500G		
PHE7	DH5α JMP62URA3ex-pTEF-Yaya*HXK1*		
**Yeast strains**			
*S. cerevisiae*	Wild-type		Culture collection of the Department of Biotechnology and Food Microbiology
*Y. yakushimensis* CBS 10253			CBS 10253
*Y. lipolytica* W29			CLIB89
*Y. lipolytica* Po1d	MatA, *leu2-270, ura3-302*^*b*^*, xpr2-322*	Leu- Ura-	CLIB139
Yl_dhxk1	*Y. lipolytica* Po1d *hxk1*	Ura-	This work
Yl_dhxk1-dglk1	Yl_dhxk1 *glk1*	–	This work
PHY118	Yl_dhxk1_dglk1 *ura3-302*^*b*^	Ura-	
PHY84-86	PHY118 pTEF-*YlHXK1*-Δloop	–	
PHY87-89	PHY118 pTEF-*YayaHXK1*		
PHY90-92	PHY118 pTEF-*ScHXK2*		
PHY93-95	PHY118 pTEF-*YlHXK1*		
PHY96-98	PHY118 pTEF-*YlHXK1-*D386E		
PHY99-101	PHY118 pTEF-*YlHXK1-*E500G		
PHY102-104	PHY118 pTEF-*YlHXK1-*D386E E500G		
PHY105-107	PHY118 pTEF-*YlHXK1-*S15A		
PHY108-110	PHY118 pTEF-*YlHXK1*-Δ7-15		
PHY111-113	PHY118 pTEF-*YlHXK1*-Δ155-182		

Growth profiles of the obtained transformants in a medium containing glucose, fructose and mixture of both were determined using microplate reader. The experiments revealed that the transformants of each genotype show very similar growth profiles. For that reason, only one strain of each genotype was used for further investigations, reducing the number of strains to 10, which growth profiles are presented in Supplementary Fig. S2.

The microculture experiment showed that *Y. lipolytica* strain expressing YlHxk1Δloop was unable to grow on neither glucose nor fructose. With that exception, overexpression of all other hexokinases restored the growth on both hexoses of the *hxk1 glk1* mutant (Supplementary Fig. S2). All the strains grew faster on glucose than on fructose.

**Figure 3:**
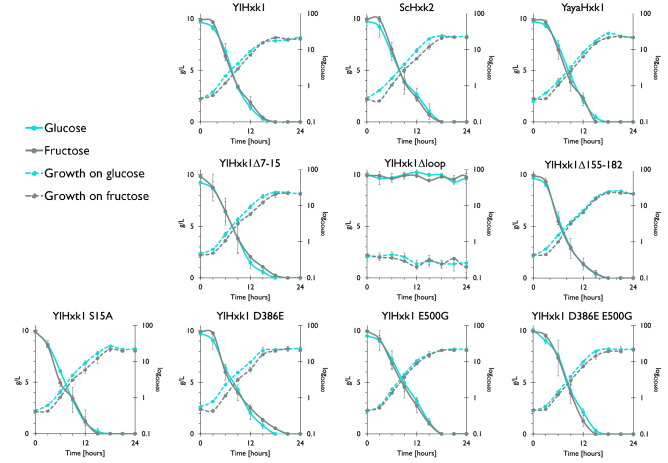
Growth and substrate utilization of *Y. lipolytica* strains expressing different hexokinase variants in YNB minimal medium with glucose or fructose.

Consistent with the growth profiles, the strain expressing YlHxk1Δloop was unable to use any of the given carbon source in monosubstrate cultures (Fig. 3). The differences in terms of growth and substrate utilization among the other transformants were only subtle.

**Figure 4:**
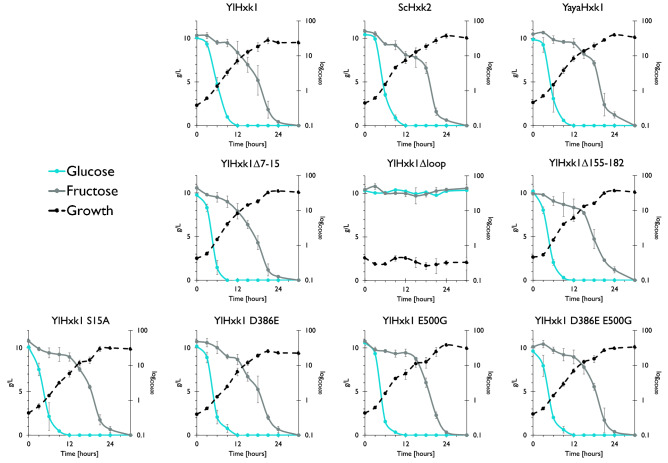
Growth and substrate utilization of *Y. lipolytica* strains expressing different hexokinase variantsin YNB minimal medium containing a mixture of glucose and fructose.

During cultivation in a medium containing a mixture of glucose and fructose, all strains (except YlHxk1Δloop, which did not grow at all) used glucose prior to fructose (Fig. 4). Some transformants were able to smoothly switch from glucose to fructose utilization (YlHxk1, YlHxk1Δ7-15, YlHxk1 D386E and YlHxk1 D386E E500G), while others showed a longer adaptation period. Interestingly, strains expressing YlHxk1Δ155-182 and YlHxk1 S15A had steep slopes of glucose utilization in the initial 3 h of cultivation. Moreover, the growth profile of the strain YlHxk1 E500G resembled diauxic growth, what could also be observed for YlHxk1 D386E E500G, YlHxk1 S15A and YlHxk1Δ155-182 strains (Fig. 4).

### Deletion of the loop in YlHxk1 results in a complete loss of hexokinase activity and hinders binding with an inhibitor

**Figure 5:**
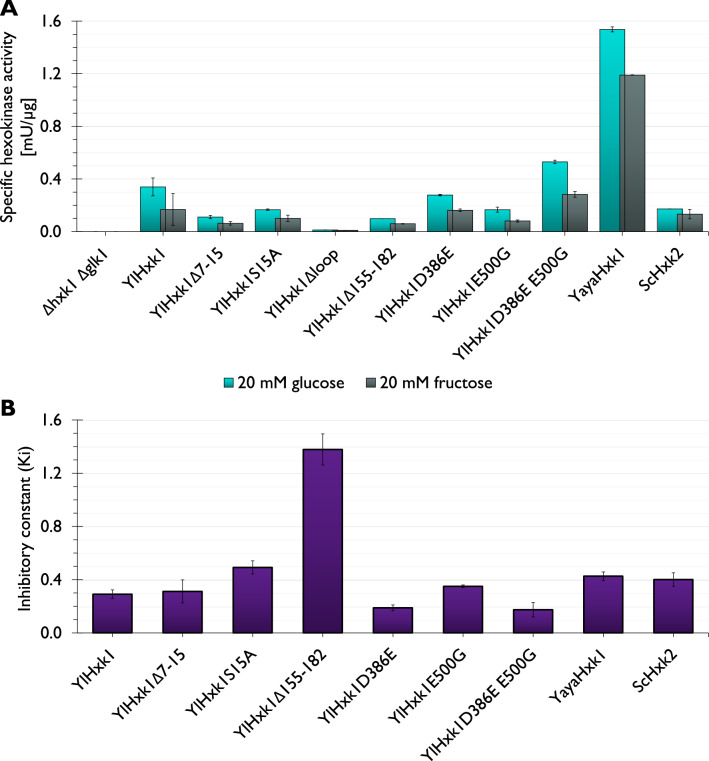
Kinetic parameters of the analyzed hexokinases. (**A**) Specific hexokinase activities; (**B**) inhibition constant values, assayed with 20 mM glucose.

The hexokinase activity measurements in the whole-cell extracts showed that deletion of the 37-amino acid loop in YlHxk1 caused complete loss of activity as for the *Y. lipolytica hxk1 glk1* strain (Fig. 5A). All other hexokinases had higher activity towards glucose than fructose. The highest activity for both substrates was shown for enzyme from *Y. yakushimensis* (YayaHxk1). Interestingly, YlHxk1 enzyme with a short loop (YlHxk1Δ155-182, mimicking YayaHxk1) was much less efficient than the native YlHxk1 with 3.4- and 2.8-fold lower activities for glucose and fructose, respectively (Fig. 5A). In general, all modifications in YlHxk1 decreased its activity, except for the double point mutation (D386E E500G), which increased the activity 1.6- and 1.7-fold for glucose and fructose, respectively, compared to the native YlHxk1.

Deletion of the amino acids 7–15 in YlHxk1 increased its affinity for glucose, with Km value over 5.5-fold lower than for native YlHxk1, a behaviour similar to the S15A substitution, however much more severe. In contrast, the same modifications decreased the affinity of the corresponding enzymes for fructose (Table 2). An opposite situation was observed for YlHxk1 E500G, with an increased affinity for fructose (2.5-fold) and a decreased affinity for glucose (about a half). Analysis of the YlHxk1 with a short loop (YlHxk1Δ155-182) showed increased Km for both substrates, what contrasted to YayaHxk1, the enzyme with naturally short loop, which was characterized by very similar Km values to YlHxk1. The highest Km fold-change for fructose was observed for ScHxk2. Due to the lack of activity, YlHxk1Δloop was not analysed for substrate affinity.

**Table 2:** Changes in affinity towards glucose and fructose of analysed hexokinases.

	Km fold-change	
	20 mM glucose	20 mM fructose
YlHxk1	1.00	1.00
YlHxk1Δ7-15	0.18 ± 0.03	3.98 ± 0.67
YlHxk1 S15A	0.55 ± 0.11	1.22 ± 0.14
YlHxk1Δ155-182	2.05 ± 0.32	2.80 ± 0.28
YlHxk1 D386E	1.05 ± 0.17	0.85 ± 0.11
YlHxk1 E500G	2.50 ± 0.24	0.53 ± 0.15
YlHxk1 D386E E500G	0.83 ± 0.21	0.87 ± 0.09
YayaHxk1	0.94 ± 0.07	1.00 ± 0.32
ScHxk2	1.22 ± 0.16	8.00 ± 0.76

Analysis of the inhibitory constant (Ki) towards the glycolysis inhibitor (T6P) showed over 4.7-fold higher value for YlHxk1 with short loop (YlHxk1Δ155-182) compared to native YlHxk1 (Fig. 5B). On the other hand, D386E and double D386E E500G mutations made YlHxk1 more sensitive to T6P.

### Overexpression of hexokinase changes substrate utilization pattern in *Y. lipolytica*

**Figure 6:**
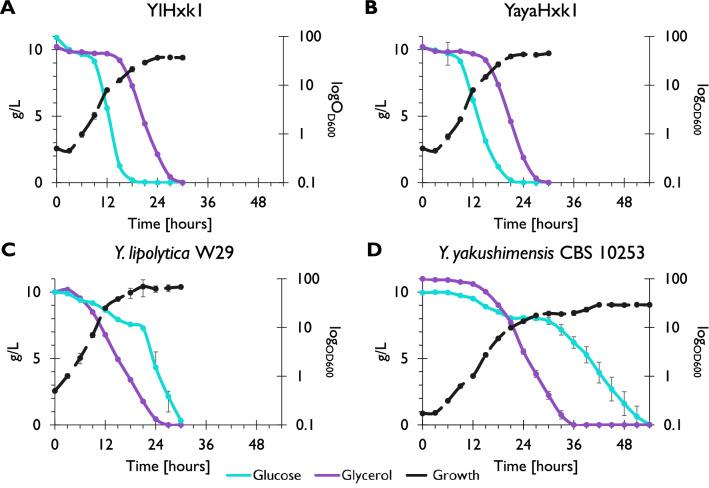
Growth and substrate utilization of *Y. lipolytica* strain expressing YlHxk1 (**A**), YayaHxk1 (**B**), a wild-type *Y. lipolytica* W29 strain (**C**) and a wild-type *Y. yakushimensis* CBS 10253 strain in YNB minimal medium with glucose and glycerol.

To further investigate the physiological role of hexokinase in *Y. lipolytica* and the function of the additional loop, we analysed the utilization of glucose and glycerol in a mixed culture by *Y. lipolytica* strains overexpressing native YlHxk1 and heterologous YayaHxk1 from *Y. yakushimensis*. The last one was used due to the lack of the additional loop. In the mixed cultures both strains preferred glucose over glycerol. Only when glucose was almost completely exhausted from the medium, glycerol utilization began (Fig. 6A,B). We also analysed the consumption of both substrates by the wild strains *Y. lipolytica* W29 and *Y. yakushimensis* CBS 10253 (Fig. 6C,D). The profile of substrate utilization showed different behaviour of these microorganisms. In contrast to the *Y. lipolytica* transformants, strains W29 and CBS 10253 consumed glucose and glycerol concomitantly from the beginning of the process, followed by rapid glycerol utilization and inhibition of glucose utilization after the logarithmic growth. Only when glycerol was almost completely exhausted, glucose utilization started again. This resulted in a second growth phase of *Y. yakushimensis* strain (39^th^ hour; Fig. 6D). Interestingly, *Y. yakushimensis* required 12- and 24 h more than *Y. lipolytica* W29 to completely utilize glycerol and glucose, respectively.

### The loop in YlHxk1 is important in gene expression regulation

**Figure 7:**
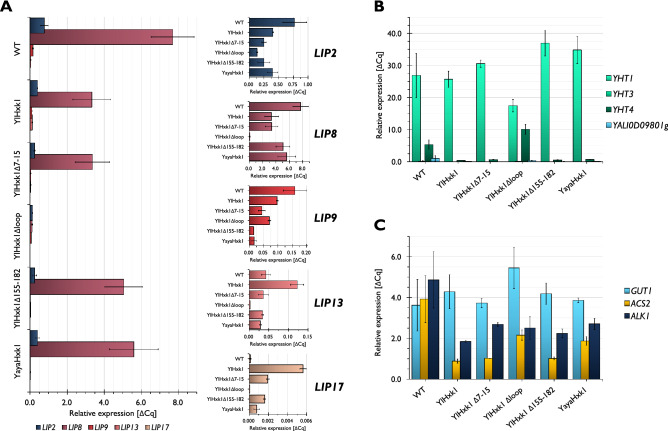
Impact of hexokinase on gene expression levels in *Y. lipolytica.* (**A**) Transcript levels of lipase-encoding genes; (**B**) expression of hexose transporters and *N*-acetylglucosamine transporter (*YALI0D09801g*); (**C**) expression of the genes involved in utilization of glycerol (*GUT1*), acetate (*ACS2*) and *n*-alkanes (*ALK1*).

Expression patterns of genes encoding lipases varied depending on the overexpressed hexokinase and in each of the analyzed strains the two most expressed genes were *LIP2* and *LIP8* (Fig. 7A). The expression patterns can be roughly divided into three sets: (1) set of *LIP2* and *LIP8*; (2) set of *LIP9* and (3) set of *LIP13* and *LIP17*. In the first one the overexpression of hexokinase caused severe repression of *LIP2* and *LIP8*. This effect was even more pronounced in a strain expressing YlHxk1 devoid of loop in which *LIP2* and *LIP8* transcript levels were 0.4- and 3.1-fold lower compared to the YlHxk1 strain, respectively and 5.9- and 70-fold lower when compared to the wild-type, respectively. In the second set consisting of *LIP9* alone the hexokinase overexpression regardless of its modification or origin also inhibited lipase expression, however the lowest ΔCq values were observed for YlHxk1Δ155-182 and YayaHxk1 strains. In contrast to the set of *LIP2* and *LIP8*, a mutant with YlHxk1Δloop did not exhibit here such strong repressive effect. Interestingly, in the case of *LIP13* and *LIP17*, which showed very weak signals, the pattern was completely different (Fig. 7A). The overexpression of native hexokinase induced expression of these genes while YlHxk1 without the loop ceased their expression almost entirely. YayaHxk1 and YlHxk1Δ155-182 had similar effect on the transcript levels of *LIP13* and *LIP17*.

The impact of hexokinase variants on the expression of the genes involved in utilization of various carbon sources was also analyzed. For hexose transporters, the three genes had three different expression patterns (Fig. 7B). The overexpression of hexokinase did not change the expression of *YHT1* except when the loop was deleted. No expression and no changes were observed for *YHT3*. In the case of *YHT4* the deletion of the loop had a strong inducing effect on its expression whereas other mutants exhibited strong repression. Additionally, the overexpression of hexokinase irrespective of its variant decreased transcript levels of the *N*-acetylglucosamine transporter (*YALI0D09801*g). Overexpression of YlHxk1 had no real effect on expression of *GUT1* (glycerol kinase) with an exception of YlHxk1Δloop which increased the expression 1.5-fold (Fig. 7C). The transcript levels of *ACS2* (acetyl-CoA synthetase) and *ALK1* (*n*-alkane inducible cytochrome P450) decreased in a result of hexokinase overexpression but in the case of *ACS2* the repression effect was less drastic in the strain overexpressing YlHxk1Δloop.

## Discussion

The present study aimed to characterize the hexokinase YlHxk1 of the yeast *Y. lipolytica* using bioinformatic as well as experimental methods. The protein was previously studied by Petit and Gancedo^13^. However, we have intended to add an in-depth understanding of its functioning by focusing on different elements of this protein. In particular, we focused on elucidating the role of the 37-amino acid loop in YlHxk1, which to the best of our knowledge is not present in any previously studied hexokinases.

The amino acid sequences alignment of YlHxk1 and other known-hexokinases, including ScHxk2 confirmed the essential difference between these enzymes—the presence of a 37-amino acid loop in YlHxk1. The additional region was also present in almost all hexokinases from the yeast belonging to the *Yarrowia* clade except for Hxk1 from *Y. yakushimensis*^28^. A phylogenetic tree constructed based on amino acid sequences of hexokinases showed that YayaHxk1, YaphHxk1 and the most protruding CahiHxk1 are the most distant proteins in relation to YlHxk1. This phylogeny does not corroborate the species tree previously published from a concatenation of 97 proteins^29^. This finding suggests that this protein in *Y. yakushimensis* has undergone a particular evolution, different from that of its counterpart in the other *Yarrowia* species.

The loop, which function is unknown, has no sequence similarities outside the *Yarrowia* clade. It prompted us to predict its structure and infer its potential functions. From the three-dimensional YlHxk1 model built using SWISS-MODEL^30^ on a *Kluyveromyces lactis* KlHxk1p hexokinase template it was found that the loop is conformationally located outside the canonical domains and seemingly takes no part in its catalytic functions. Yet again, the structure of this loop could not be assigned to any known protein structures by the SWISS-MODEL software. In turn, the YayaHxk1, compared to the other proteins from the *Yarrowia* clade, has much shorter fragment consisting of only 9 instead of 37 amino acids. The predicted model of YayaHxk1 revealed that this short sequence forms an α-helix, which is not present in YlHxk1 (Fig. 2B). This might stem directly from the chemical nature of the amino acids forming the short loop in YayaHxk1. Similar structure could be obtained by deleting the 155–182 region in YlHxk1 and substituting the sequence ^148^AHE^150^ to ^148^ESS^150^ as in YayaHxk1 (Supplementary Fig. S3).

The bioinformatic analyses delivered insights into the potential impact of the 37-amino acid loop on the functioning of YlHxk1, which deserved to be verified experimentally. Therefore, we prepared a *Y. lipolytica* strain deprived of hexose kinases (*hxk1 glk1*) which was the basis for constructing strains expressing various variants of YlHxk1 and heterologous hexokinases. Except for a strain expressing hexokinase missing the loop (YlHxk1Δloop), all other modifications restored the growth of the corresponding strain on both glucose and fructose. The former modification produced a protein with no hexokinase activity. These results were confirmed by hexokinase activity measurement using whole cell extracts. The protein with loop deletion showed no kinase activity neither for glucose nor for fructose. As this structure is conformationally located outside the active site, we hypothesize that the loop removal causes changes in the enzyme conformation, rendering the protein unable to bind glucose/fructose, ATP or both, what in turn leads to loss of its catalytic function.

The highest hexokinase activity was observed for the *Y. lipolytica* strain expressing hexokinase from *Y. yakushimensis*. At the same time, the affinities of YayaHxk1 for both glucose and fructose did not differ from the obtained for strain with YlHxk1. Significantly increased activity compared to YlHxk1 (over 4.5- and 7-fold, for glucose and fructose, respectively) might stem from the environmental adaptation of *Y. yakushimensis*. This yeast species has only been isolated from a gut of a termite^31^. Because termites consume dead plants at any level of decomposition^32^, its microbiome must be adapted to breakdown cellulose^33^, what in turn generates substantial amount of monosaccharides. It could be hypothesized that high activity of YayaHxk1 is advantageous for growth of *Y. yakushimensis* in high-sugar concentration environment. As the shorter loop in YayaHxk1 provided a clear improvement in hexokinase activity compared to YlHxk1, it could be expected that YlHxk1 mimicking YayaHxk1 (YlHxk1Δ155-182) would behave in the similar manner. However, that was not the case; alteration in the loop region of YlHxk1 caused severe decrease in activity and affinity for both glucose and fructose. Despite that, it has to be noted that the loop in YayaHxk1 differs from YlHxk1 not only in length, but also in the amino acid residues. It is possible that conformation induced by these residues might positively affect the catalytic activity of YayaHxk1, however, as mentioned above, this idea needs to be verified experimentally.

The strain expressing YlHxk1 with E500G mutation grew similarly on both glucose and fructose. As evidenced by the fold-change in Km compared to the native enzyme, better growth and fructose utilization was a direct effect of increased affinity towards this hexose. On the other hand, this modification caused over twofold decrease in activity for both glucose and fructose compared to the unmodified protein. It was previously reported this mutation in ScHxk2 severely affects catalytic activity and substrate recognition^24^.

After determining kinetic parameters of hexokinases we set to analyse the impact of introduced modifications on the interactions with inhibitor. T6P is an intermediate in the biosynthesis of trehalose from glucose 6-phosphate and UDP-glucose and functions as a regulator of glycolysis in yeast, filamentous fungi and plants^17,34–36^. Petit and Gancedo^13^ reported that YlHxk1 is extremely sensitive to inhibition by T6P. In this study we wanted to point out the element of YlHxk1 that evokes this sensitivity. As YlHxk1Δloop lacked activity on glucose and fructose, it was impossible to assess its interaction with T6P. Instead, we used the strain expressing YlHxk1 with a short loop (YlHxk1Δ155-182) that could partially substitute for YlHxk1Δloop in this experiment. YlHxk1Δ155-182 showed significantly higher Ki towards T6P indicating that the loop plays an important role in the enzyme-inhibitor interaction. The S15A substitution also increased Ki, however, much less dramatically than in the case of YlHxk1Δ155-182. The S15A mutation in ScHxk2 causes impairments in phosphorylation and dimerization^25^. On the other hand, the D386E point mutation increased the sensitivity towards T6P. In *S. cerevisiae* Hxk2, the consensus amino acids D386 and E500 were associated with derepression of *SUC2* gene by glucose as seen in *ScHXK2* null mutant, as well as with impairments in catalytic activity and substrate recognition^24^. However, these mutations have not been associated with T6P.

Following the inhibitory effect of T6P on glycolysis, we hypothesized, that *Y. lipolytica* may require very strict control of the metabolism at this point and that the very high sensitivity of YlHxk1 to T6P may be the way to slow down the incorporation of glucose when alternative carbon source is present in the medium. In line with this hypothesis, the qRT-PCR analysis showed that *TPS1* gene, encoding T6P synthase^37^, was less expressed during glucose utilization in the wild-type strain (Supplementary Fig. S4). Wild strains of *Y. lipolytica* prefer glycerol over glucose^9,21^. However, overexpression of either YlHxk1 or YayaHxk1 reversed the order of substrate consumption. This may be the result of the amount of hexokinase in the cell, which cannot be inhibited by T6P anymore. We also checked the behaviour of the wild *Y. yakushimensis* strain in the medium with mixture of glucose and glycerol. At first, this species used both substrates simultaneously, followed by inhibition of glucose utilization. Only after glycerol was exhausted from the medium, glucose could be used again. We speculate that this phenomenon may be also partially connected to the differences in the hexokinase structure. However, this hypothesis has to be further investigated. Nonetheless, our research showed a relationship between the loop in YlHxk1 and the inhibition of this protein by T6P.

Apart from the catalytic function, hexokinases also contribute to the regulation of gene expression as a crucial component of the main glucose repression pathway in *S. cerevisiae* as reviewed in^38^. The YlHxk1 was previously reported to act in a similar manner to ScHxk2 by repressing *LIP2* gene encoding extracellular lipase^20^, as well as influencing expression of genes involved in tricarboxylic acid cycle and erythritol biosynthesis^9^. Additionally, YlHxk1 successfully substituted ScHxk2 in repression of the invertase-encoding gene (*SUC2)* in *S. cerevisiae*^13^. The moonlighting proteins such as hexokinase, acting in different cellular compartments, contain certain signal sequence(s) that allow for their transport e.g. from the cytoplasm to the nucleus where the repressor/activator complexes are assembled^26,39^. The literature data indicates that approximately 10% of ScHxk2 is located in the nucleus and that this localization is important for glucose catabolite repression^40^. A sequence of two positively charged amino acids flanking three residues, one of which is proline may act as NLS. Petit and Gancedo^13^ identified four such sequences in YlHxk1: ^8^KPPSR^12^, ^281^KDIPK^285^, ^314^KVLPR^318^ and ^527^KPGVK^513^. The bioinformatic tools, such as SeqNLS^41^ allows to predict NLS using primary protein sequence. According to SeqNLS, YlHxk1p presents only one NLS sequence, ^3^HLGPRKPPSRK^13^ (Supplementary Fig. S1) which shows 66.67% identity to the decapeptide ^7^KKPQARKGSM^16^, important for nuclear localization of ScHxk2^40^. In the current study, we verified if the ^3^HLGPRKPPSRK^13^ fragment of YlHxk1 sequence acts as a NLS. Therefore, we checked the impact of the introduced mutations on the expression of genes encoding lipases and genes responsible for utilization of various carbon sources. Consistent with the study of Fickers et al.^20^, overexpression of *YlHXK1* caused almost twofold reduction of *LIP2* expression confirming hexokinase influence on gene expression in *Y. lipolytica*. The lowest *LIP2* expression were observed in strain expressing YlHxk1Δloop while the transcript level in a strain expressing YlHxk1 without suspected NLS was not that much decreased. Similar behaviour was observed for the *LIP8* gene expression. Expression of the other analyzed lipases was very low in all strains, however, interesting pattern could be seen for *LIP13* and *LIP17* as *YlHXK1* overexpression induced expression of these genes. For the 7–15 region the obtained result is counter-intuitive. Hexokinase without the NLS should be unable to get into the nucleus. Therefore, expression of the genes including *LIP2* and *LIP8* could be expected at least at the level of wild-type strain or even elevated. That is the opposite to what we observed in our experiment and due to that we cannot state that ^3^HLGPRKPPSRK^13^ is the NLS in YlHxk1.

YlHxk1 without the loop had a strong repressive effect on the *YHT1* gene encoding major hexose transporter. Surprisingly, opposite behaviour was observed for *YHT4*-encoding gene. This observation suggests different regulatory networks playing role in expression of these genes and hexokinase being their important component. To the best of our knowledge, this is the first time the influence of hexokinase on expression of hexose transporter genes was analyzed in *Y. lipolytica*. In the model yeast *S. cerevisiae* the transcriptional regulation of hexose transporters occurs via Rgt1-mediated pathway which does not include hexokinase^42^. Furthermore, from our data it transpires that YlHxk1 plays a role in regulation of expression of other genes including *ACS2*, *ALK1* and *GUT1*.

Based on the presented results we hypothesize that hexokinase devoid of the loop is easier to transport through the nuclear pore or bind more easily with other proteins forming the repressor complex (e.g. Mig1). It might also be possible that the loop takes part in the recruitment of the transcription machinery. In *S. cerevisiae,* the main hexokinase (ScHxk2) directly interacts with Mig1 in a cluster with DNA fragments containing the MIG1 binding site e.g. of *SUC2* promoter^39^. Similar interaction may occur in *Y. lipolytica*, however, further experiments, such as localization study using GFP-fused proteins or yeast two hybrid system, are required. Interestingly, YayaHxk1 successfully substituted for YlHxk1 in repression of most of the genes.

## Conclusion

The presented work gives valuable insights into the understanding of hexokinase functioning and more broadly, on sugar assimilation in *Y. lipolytica*. A substantial progress was achieved by showing that the 37-amino acid extra-loop in the central region of YlHxk1 is essential for activity of this protein and through that on growth on glucose and fructose. Furthermore, hexokinase from *Y. yakushimensis* (YayaHxk1) containing shorter loop also allows for normal growth, with a much higher enzymatic activity of the corresponding *Y. lipolytica* transformant on glucose and fructose. Alterations of the loop region and in other amino acids induce a panel of changes in hexokinase activity, affinity towards glucose and fructose as well as sensitivity to T6P. The overexpression of YlHxk1 changes the peculiar pattern of substrate utilization in *Y. lipolytica* allowing glucose being used more preferably than glycerol. Additionally, our study reveals that overexpression of YlHxk1 and its different variants interferes with the expression of genes encoding lipases, hexose transporters and genes encoding proteins involved in utilization of alternative to glucose or fructose carbon sources. Moreover, the loop region appears to be an important element of YlHxk1 implicated in its regulatory function.

## Methods

### Media and culture conditions

The strains of *Y. lipolytica* and *Y. yakushimensis* were routinely maintained in YPD medium consisting of 10 g/L yeast extract, 10 g/L peptone, 20 g/L glucose or glycerol with 10 g/L agar (for plates) at 28 ^o^C. Minimal (YNB) medium for selection of the *Y. lipolytica* transformants was prepared using 1.7 g/L yeast nitrogen base (without amino acid and ammonium sulfate, Sigma-Aldrich, Saint Louis, MI), 10 g/L glycerol, 5 g/L NH_4_Cl, 50 mM phosphate buffer pH 6.8 with 10 g/L agar. The *Escherichia coli* strains harboring plasmids were cultured overnight in LB medium (5 g/L yeast extract, 10 g/L triptone, 10 g/L NaCl with 10 g/L agar in plates and 0.05 mg/L kanamycin) at 37 °C. For long-term storage the strains were kept at − 80 °C in 500 g/L glycerol.

### Plasmid preparation

In this study, two types of plasmids were used. Gene disruption-carrying cassettes were constructed using pCR-Blunt II TOPO vector (Invitrogen, Carlsbad, CA), whereas plasmids used for gene overexpression were based on a JMP62 plasmid^19^. All genomic sequences were retrieved from GRYC database (http://gryc.inra.fr/).

The disruption cassettes were prepared as described in^27^. Briefly, 1 kb fragments representing promoter (P) and terminator (T) sequences of *YlHXK1* (*YALI0B22308g*) and *YlGLK1* (*YALI0E15488g*) were amplified by PCR from *Y. lipolytica* W29 genomic DNA and subsequently fused using PCR-fusion technique. The obtained PT fragments were cloned into pCR-Blunt II TOPO vector. Resulting plasmids were then digested with I-*Sce*I restriction enzyme and the I-*Sce*I digested *URA3ex* (U) or *LEU2ex* (L) marker was inserted to obtain the disruption cassettes.

The overexpression cassettes were constructed by PCR amplification of hexokinase from the genomic DNA of *Y. lipolytica* W29 and genomic DNA of *Y. yakushimensis* CBS 10253. The mutated versions of *Y. lipolytica* hexokinase were also prepared and include: YlHxk1Δ7-15, YlHxk1Δloop, YlHxk1Δ155-182, YlHxk1 S15A, YlHxk1 D386E, YlHxk1 E500G, YlHxk1 D386E E500G. Annotated DNA sequence of *YlHXK1* is presented on Supplementary Fig. S1. Obtained PCR fragments with the appropriate restriction enzyme sites were then digested and ligated into *Bam*HI/*Bgl*II and *Xma*JI (*Avr*II) digested JMP62 vector carrying a strong constitutive TEF promoter. All constructs were verified using PCR and DNA-sequencing (Genomed S.A, Warsaw, Poland).

### Strain construction

Prior to transformation, the strains were grown overnight on YPD plates. The whole inoculation loop of biomass was suspended in 100 µL of transformation solution consisting of 125 µL of 500 g/L PEG, 6.25 µL of 2 M DTT and 6.25 µL of 2 M LiAc containing 10 µL of *Not*I digested disruption or overexpression cassettes (500 ng of DNA) and 5 µL carrier DNA (10 mg/mL; Invitrogen, Carlsbad, CA). Prepared cell suspensions were mixed using vortex and incubated at 28 °C for 30 min. Subsequently, the samples were mixed again, incubated at 42 °C for 10 min, plated onto selection media (YNB with glycerol) and incubated at 28 °C for 48 h. The obtained transformants were verified by PCR on their genomic DNA extracted as in^43^ with appropriate primers (JMP62-pTEF-START and reverse primers for gene amplification). The primers used in this study are listed in Supplementary Table S2 and the strains are presented in Table 1.

### Microcultures

The growth profiles of *Y. lipolytica* strains growing on glucose, fructose and a mixture of both sugars were obtained using a Synergy H1 microplate reader (BioTek Instruments, Winooski, VT). Prior to the experiment, the cells were grown overnight in 5 mL YPD, washed thrice with sterile distilled water to remove any YPD residuals and standardized to OD_600_ = 10. The cultures were carried out in 96-well microtiter plates (NEST, Wuxi, China) with a working volume of 200 µL, 600 rpm linear shaking and initial OD_600_ of 0.5. The media consisted of 1.7 g/L YNB, 5 g/L NH_4_Cl, 50 mM phosphate buffer pH 6.8 and 10 g/L of glucose, or fructose. The temperature was maintained at 28 °C throughout the process. The growth was monitored by measuring optical density in 30 min intervals. Experiments were conducted in three biological replicates.

### Substrate utilization kinetics

The kinetics of hexose utilization was performed in 250 mL Erlenmeyer flasks with 50 mL of medium composed of 1.7 g/L YNB, 5 g/L NH_4_Cl, 50 mM phosphate buffer pH 6.8 and 10 g/L of glucose, fructose or mixture of both. The inoculum was prepared as in the *Microcultures* subsection. The initial OD_600_ was set up at 0.5 and cultures were incubated at 28 °C on a rotary shaker with 180 rpm shaking speed. Experiments were conducted in three biological replicates. The samples were taken every 3 h. The growth was monitored by measuring optical density at 600 nm using SmartSpec Plus spectrophotometer (Biorad, Hercules, CA). Monosaccharides concentration was determined using Dionex UltiMate 3000 HPLC instrument (Dionex-Thermo Fisher, UK) equipped with a Carbohydrate H + column (Thermo Scientific, Waltham, MA) coupled to an UV (λ = 210 nm) and RI (Shodex, Ogimachi, Japan) detectors. The column was eluted with 25 mM trifluoroacetic acid (TFA) at 65 °C and a flow rate of 0.6 mL/min.

Kinetics of glucose and glycerol utilization by *Y. yakushimensis* and *Y. lipolytica* transformants overexpressing native *YlHXK1* and a heterologous *YayaHXK1* in a mixed culture were investigated as described above, with 10 g/L of glucose and 10 g/L of glycerol. Experiments were performed in three biological replicates.

### Preparation of yeast whole-cell extracts

A volume of 50 mL of YNB medium with glycerol in 250 mL Erlenmeyer flasks were inoculated to OD_600_ = 0.5 with biomass prepared as in the *Microcultures* section. The cultures were carried out at 28 °C and 180 rpm for 24 h. The cells were then centrifuged (4500 rpm/5 min/4 °C) and resuspended in an ice-cold PBS buffer pH 7.4. Next, the suspensions were standardized to OD_600_ = 1 using PBS, transferred to 2 mL Eppendorf tubes with 1/3 volume glass beads (425–600 µm diameter) and homogenized using BeadBug Microtube Homogenizer (Benchmark Scientific, Edison, NJ) at 4000 rpm for 1 min. The procedure was repeated thrice with 2 min incubations on ice between homogenizations. Whole-cell extracts prepared like so were then analysed for protein concentration using Bradford assay^44^ and standardized to a concentration of 30 µg/mL with PBS for further analyses.

### Hexokinase activity assay and Michaelis constant determination

Hexokinase activity in the standardized whole-cell extracts (30 µg/mL protein) was measured with 20 mM glucose or fructose using Hexokinase Colorimetric Assay Kit (Merck, Darmstadt, Germany) according to the supplied protocol and Synergy H1 microplate reader (BioTek). The Michaelis constants (Km) for glucose and fructose were determined by measuring hexokinase activity as described above using 5, 10, 15 and 20 mM of the appropriate hexose. The experiments were performed in three biological replicates.

### Trehalose-6 phosphate inhibition assay

The inhibition constants were determined by measuring hexokinase activity in the standardized whole-cell extracts (30 µg/mL protein) with 20 mM glucose as described above with the addition of 0.5, 1, 2.5 and 5 mM trehalose 6-phosphate (Cayman Chemical, Ann Arbor, MI). The inhibition assays were performed in three biological replicates.

### Gene expression analysis

*Yarrowia lipolytica* mutants were cultivated in 250 mL Erlenmeyer flasks with 50 mL of medium composed of 1.7 g/L YNB, 5.1 g/L NH_4_Cl, 50 mM phosphate buffer pH 6.8 and 2 g/L of glycerol to a mid-exponential growth phase (12 h). From the collected samples, RNA was immediately extracted using Total RNA Mini kit (A&A Biotechnology, Gdynia, Poland) according to the supplied protocol and its concentration and quality was verified using Biochrom WPA Biowave DNA spectrophotometer (Biochrom Ltd., Cambridge, UK) Extracted RNA samples were treated with DNase (A&A Biotechnology, Gdynia, Poland) and reverse transcribed to cDNA using Maxima First Strand cDNA Synthesis Kit for RT-qPCR (Thermo Scientific). The obtained cDNA samples were then used for qPCR reaction using Maxima SYBR Green qPCR Master Mix (Thermo Scientific) and primers listed in Table S2 in the PCRmax Eco 48 thermal cycler (Illumina, San Diego, CA). The expression of analyzed genes was standardized to the expression of actin (*YlACT1, YALI0D08272g*) gene. The gene expression levels were examined in three biological replicates.

### Bioinformatic methods

The amino acid sequences of YlHxk1, ScHxk1, ScHxk2 and *K. lactis* hexokinase (KlHxk1) were retrieved from the GRYC (http://gryc.inra.fr/) and NCBI databases^45^. The sequences of the hexokinases from the yeast species of the *Yarrowia* clade are available in Supplementary File S1; they have been predicted from the genome sequence published by Červenak et al.^29^. All the amino acid sequences are numbered starting from the initiator methionine. The sequences were compared using Blastp^46^, aligned with ClustalOmega^47^ and the resulting alignments were visualized using JalView 2.11.1.0^48^. The three-dimensional models of hexokinases were generated using SWISS-MODEL^30^ and visualized with PyMOL (The PyMOL Molecular Graphics System, Version 2.4.0, Schrödinger, LLC). A phylogenetic tree for hexokinases was constructed by using the Maximum Likelihood method with the JTT matrix-based model. A discrete Gamma distribution was used to model evolutionary rate differences among sites (4 categories (+G, parameter = 0.9178)). The rate variation model allowed for some sites to be evolutionarily invariable ([+I], 14.63% sites). All positions containing gaps were removed. There were a total of 484 positions in the final dataset. Bootstrap values were calculated from 100 replicates. Evolutionary analyses were conducted in MEGA7^49^. The potential NLS in YlHxk1 were predicted using SeqNLS^41^.

## Supplementary Information

Supplementary Information
