# Supplementary material for: A 37-amino acid loop in the Yarrowia lipolytica hexokinase impacts its activity and affinity and modulates gene expression
Source: Sci Rep. 2021 Mar 19;11:6412. doi: 10.1038/s41598-021-85837-8 (PMC7979807; doi:10.1038/s41598-021-85837-8)
Supplement: Supplementary file 1 — Supplementary Information [file 41598_2021_85837_MOESM1_ESM.pdf]

SUPPLEMENTARY INFORMATION

A 37-amino acid loop in the *Yarrowia lipolytica* hexokinase impacts its activity and affinity and modulates gene expression

Piotr Hapeta[1], Patrycja Szczepańska[1], Cécile Neuvéglise[2] and Zbigniew Lazar[1,*]

[1]Wrocław University of Environmental and Life Sciences, Faculty of Biotechnology and Food Science, Department of Biotechnology and Food Microbiology, Chełmońskiego 37, 51-630 Wrocław, Poland

[2]SPO, INRAE, Montpellier SupAgro, Univ Montpellier, 34060 Montpellier, France

*zbigniew.lazar@upwr.edu.pl

Fig. S1

YlHXK1 (YALI0B22308g) (2041 bp)

ATGGTTCATCTTGGTCCCCGAAAACCCCCGTCCCGAAAGGTGAGTATGATTGCACATTAGGGGGATGAGCGATTAGCGACATGCAAAGACAACATGGCATAGCGGTA

M V H L G P R K P P S R K

R7-S15 (NLS)

ORF Intron

20 40 60 80 100

TTACAGGGAGCTCCACAGATACACGGTTCTGGCGAAAACAGATACACGGCTCCGATGGTGCAAGGACGTGATAGTAAACAGACGTCCAAGTCGTCGCCTTGGAACAT

Intron

120 140 160 180 200

GACGAGTCATACCCTCACACAACCACCCTGTCACGTCCAGCACGCCGTCGGACATGACTTCCGTCTGTGTTGATTCACAGCCATTGTGAGGGATGTGATTGGTGCTG

Intron

220 240 260 280 300 320

CGACATGGAAGGAAAGGTGACTGTCTACCGTTGCCACCGGTTTGGTTCTGTACACGTTCGGTGTCCAAGTAATAGCCACCTTGTCACATGCTGTTGCCTGCAGGATG

Intron

340 360 380 400 420

TTGTCTTCGCTCGCCAGAAAAGGCAACTCGCAGCCATACTAACACAGGGCTCAATGGCAGACGTCCCGCGGGACCTGCTGGAGCAAATCTCCCAGCTTGAAACCATC

G S M A D V P R D L L E Q I S Q L E T I

Intron ORF

440 460 480 500 520

TTCACCGTTTCGCCCGAAAAGCTGCGTCAAATCACCGACCACTTTGTGTCCGAGCTCGCTAAAGGCCTCACAAAGGAGGGTGGAGATATCCCCATGAACCCCACCTG

F T V S P E K L R Q I T D H F V S E L A K G L T K E G G D I P M N P T W

ORF

540 560 580 600 620 640

GATTCTGGGATGGCCCACCGGAAAGGAGAGCGGCTGCTATCTGGCTCTCGACATGGGTGGCACCAACCTGCGAGTTGTCAAGGTGACTCTGGACGGCGACCGAGGCT

I L G W P T G K E S G C Y L A L D M G G T N L R V V K V T L D G D R G

ORF

660 680 700 720 740

TCGACGTCATGCAGTCCAAGTACCACATGCCCCCCAACATCAAGGTCGGCAAGCAAGAGGAGCTGTGGGAGTACATTGCCGAATGTCTGGGCAAGTTCTTGGCCGAC

F D V M Q S K Y H M P P N I K V G K Q E E L W E Y I A C L G K F L A D

ORF

760 780 800 820 840

YlHXK1 (YALI0B22308g) (2041 bp) (from 857-1712 bp)

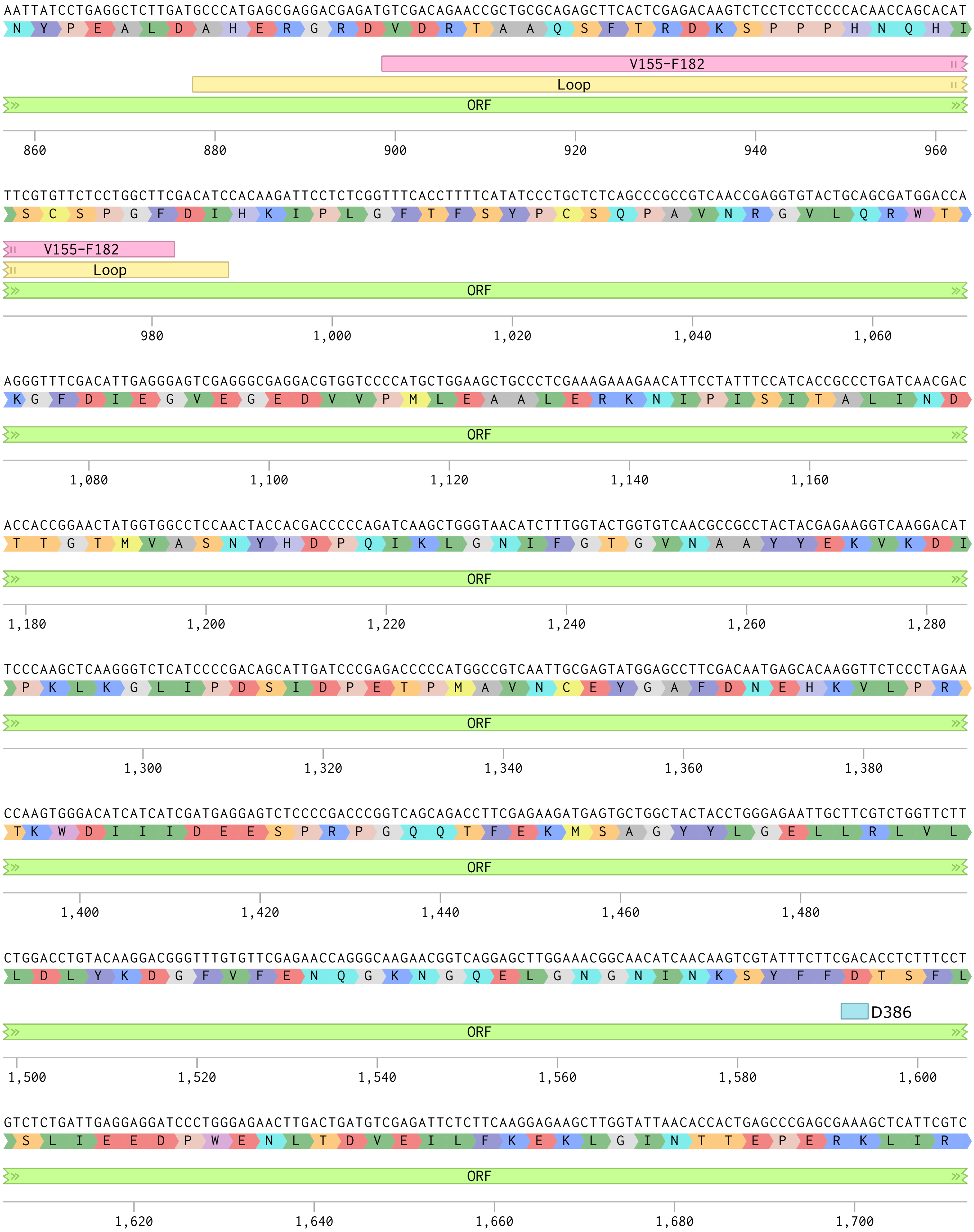

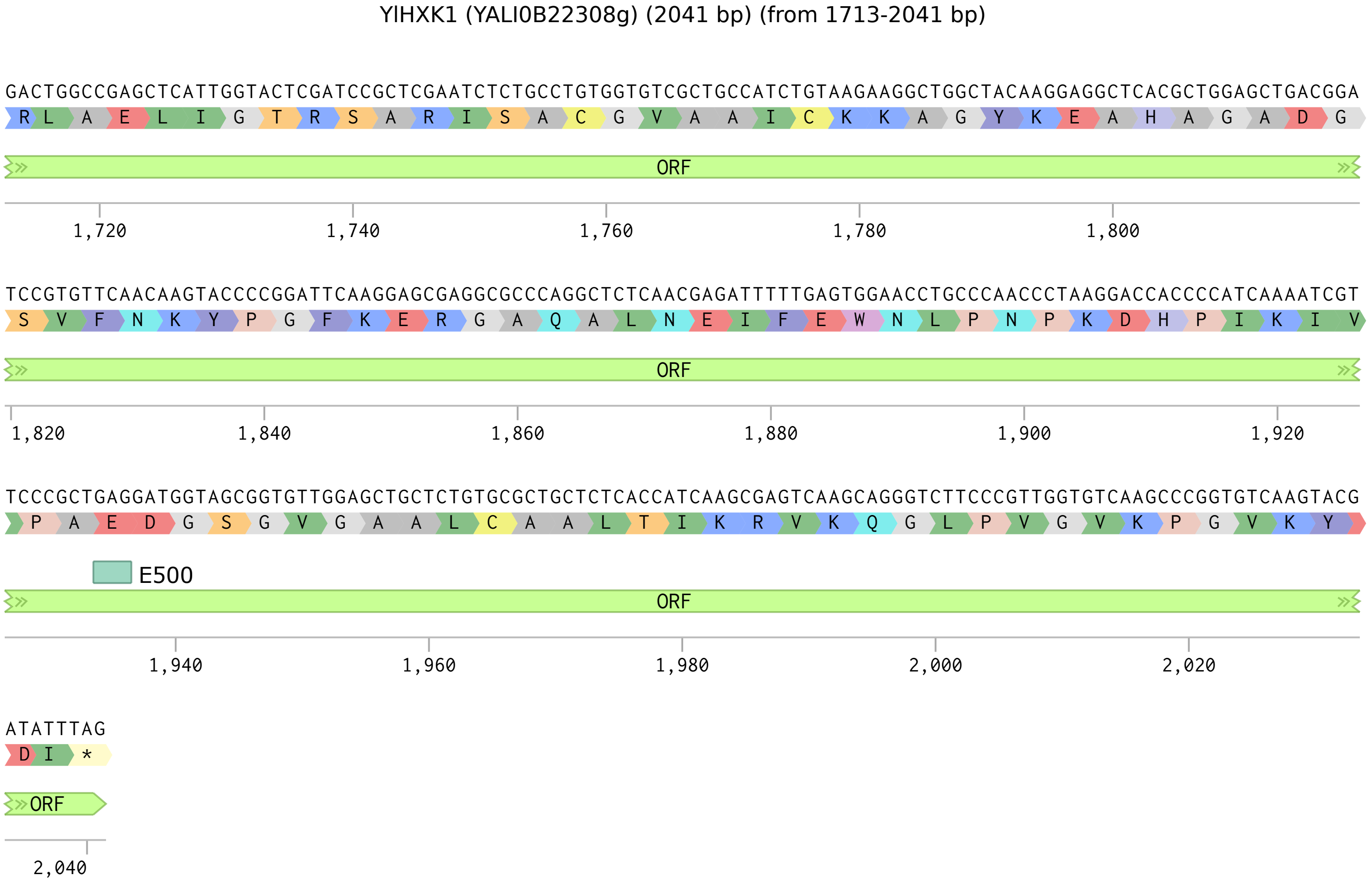

Figure S1. Sequence of Y. lipolytica hexokinase (YALI0B22308g).

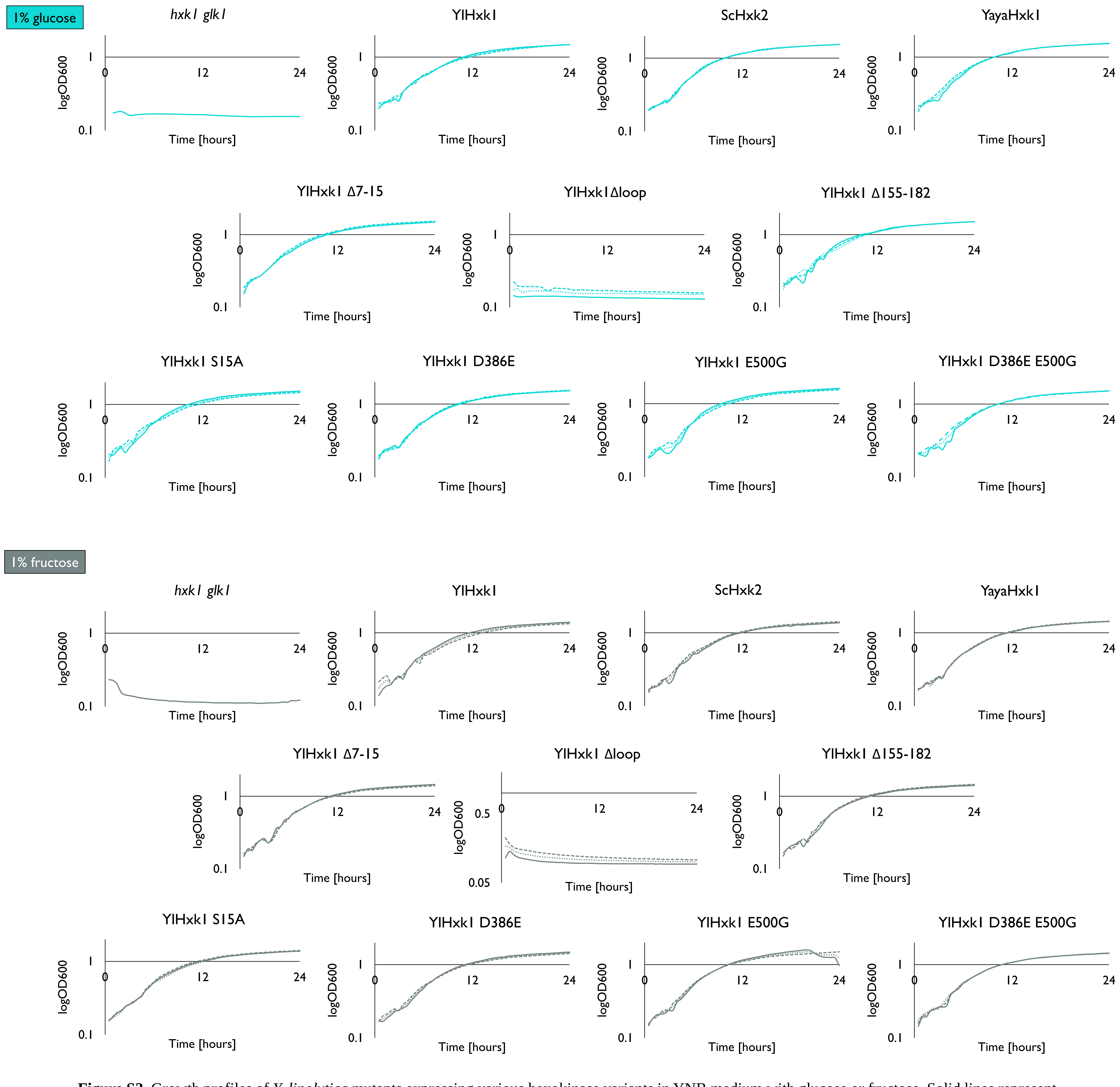

Figure S2. Growth profiles of *Y. lipolytica* mutants expressing various hexokinase variants in YNB medium with glucose or fructose. Solid lines represent growth of strains selected for further experiments.

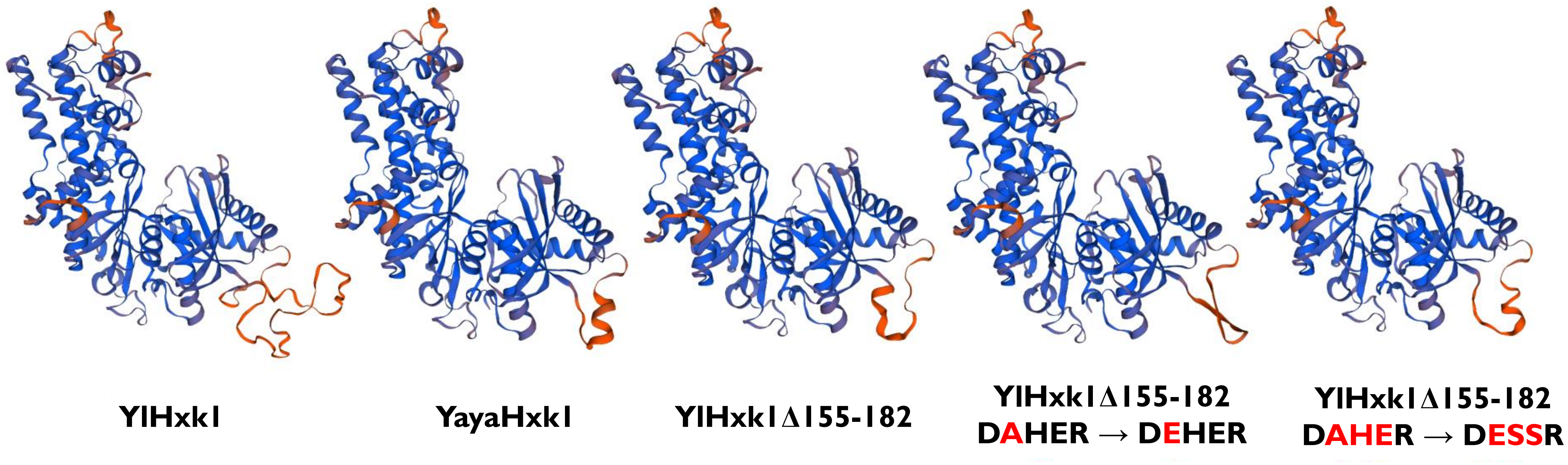

Figure S3. Implications of the mutations in the loop region of YlHxk1 on its conformation.

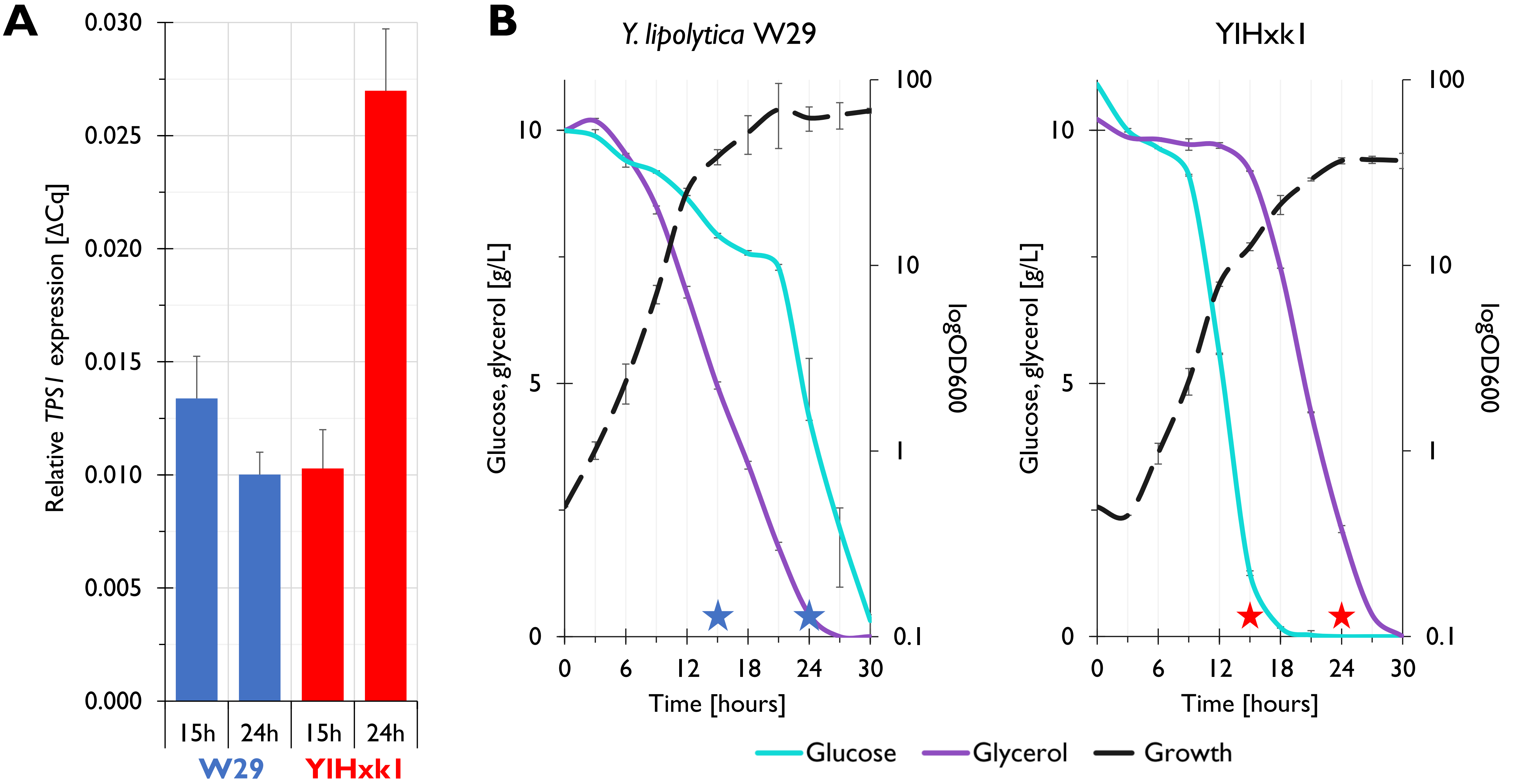

Figure S4. Trehalose phosphate synthase gene (*TPS1*) expression levels (A) in a wild-type *Y. lipolytica* W29 and a *Y. lipolytica* overexpressing YlHxk1 during growth in YNB medium with a mixture of glycerol and glucose (B). Blue and red stars indicate RNA sampling time.

Table S1. Genetic distances between Hxk1p from the *Yarrowia* clade

**Identity**	YlHxk1p	Yake_Hxk1p	Yaos_Hxk1p	Yapo_Hxk1p	Yade_Hxk1p	Yabu_Hxk1p	Yadi_Hxk1p	Yaga_Hxk1p	Yaho_Hxk1p	Yaal_Hxk1p	Yaph_Hxk1p1	Yaya_Hxk1p	Cahi_Hxk1p
YlHxk1p	534	99%	98%	98%	98%	97%	97%	97%	97%	96%	92%	89%	74%
Yake_Hxk1p	531	534	98%	98%	98%	97%	97%	97%	97%	96%	92%	89%	74%
Yaos_Hxk1p	528	527	534	98%	97%	97%	97%	97%	96%	96%	92%	89%	74%
Yapo_Hxk1p	527	527	527	534	97%	98%	97%	97%	97%	95%	92%	90%	74%
Yade_Hxk1p	524	524	521	520	534	97%	99%	97%	96%	95%	91%	88%	74%
Yabu_Hxk1p	523	523	523	524	519	534	97%	96%	96%	95%	91%	89%	74%
Yadi_Hxk1p	523	523	520	519	531	518	534	98%	96%	96%	92%	88%	74%
Yaga_Hxk1p	523	523	523	521	523	515	526	534	96%	96%	92%	88%	74%
Yaho_Hxk1p	520	518	517	521	513	516	514	517	533	96%	92%	89%	74%
Yaal_Hxk1p	513	513	513	512	510	511	513	513	513	534	92%	88%	74%
Yaph_Hxk1p1	493	493	493	496	491	491	492	493	493	492	534	86%	75%
Yaya_Hxk1p	479	480	476	481	474	477	474	474	476	471	462	506	72%
Cahi_Hxk1p	401	401	401	403	402	402	404	403	399	401	405	389	537

**Similarity**	YlHxk1p	Yake_Hxk1p	Yaos_Hxk1p	Yapo_Hxk1p	Yade_Hxk1p	Yabu_Hxk1p	Yadi_Hxk1p	Yaga_Hxk1p	Yaho_Hxk1p	Yaal_Hxk1p	Yaph_Hxk1p1	Yaya_Hxk1p	Cahi_Hxk1p
YlHxk1p	0	99%	99%	99%	99%	99%	99%	99%	98%	97%	97%	92%	84%
Yake_Hxk1p	533	0	99%	99%	99%	99%	98%	99%	98%	97%	97%	92%	84%
Yaos_Hxk1p	532	531	0	99%	99%	99%	99%	99%	98%	97%	97%	92%	85%
Yapo_Hxk1p	532	532	532	0	99%	99%	99%	99%	99%	97%	97%	92%	85%
Yade_Hxk1p	530	529	530	530	0	98%	99%	98%	98%	97%	97%	92%	85%
Yabu_Hxk1p	530	529	530	531	528	0	98%	98%	99%	97%	96%	92%	84%
Yadi_Hxk1p	529	528	529	529	533	527	0	99%	98%	97%	97%	92%	85%
Yaga_Hxk1p	530	530	532	530	528	528	529	0	98%	97%	97%	92%	85%
Yaho_Hxk1p	528	528	527	529	525	529	526	527	0	97%	97%	92%	84%
Yaal_Hxk1p	520	519	519	521	519	520	520	519	523	0	96%	91%	84%
Yaph_Hxk1p1	519	519	518	520	518	517	519	518	519	516	0	91%	84%
Yaya_Hxk1p	494	493	494	495	492	493	492	494	493	488	487	0	81%
Cahi_Hxk1p	458	457	459	460	460	456	461	460	456	457	455	439	0

**Gap**	YlHxk1p	Yake_Hxk1p	Yaos_Hxk1p	Yapo_Hxk1p	Yade_Hxk1p	Yabu_Hxk1p	Yadi_Hxk1p	Yaga_Hxk1p	Yaho_Hxk1p	Yaal_Hxk1p	Yaph_Hxk1p1	Yaya_Hxk1p	Cahi_Hxk1p
YlHxk1p	0	0%	0%	0%	0%	0%	0%	0%	0%	0%	0%	5%	1%
Yake_Hxk1p	0	0	0%	0%	0%	0%	0%	0%	0%	0%	0%	5%	1%
Yaos_Hxk1p	0	0	0	0%	0%	0%	0%	0%	0%	0%	0%	5%	1%
Yapo_Hxk1p	0	0	0	0	0%	0%	0%	0%	0%	0%	0%	5%	1%
Yade_Hxk1p	0	0	0	0	0	0%	0%	0%	0%	0%	0%	5%	1%
Yabu_Hxk1p	0	0	0	0	0	0	0%	0%	0%	0%	0%	5%	1%
Yadi_Hxk1p	0	0	0	0	0	0	0	0%	0%	0%	0%	5%	1%
Yaga_Hxk1p	0	0	0	0	0	0	0	0	0%	0%	0%	5%	1%
Yaho_Hxk1p	1	1	1	1	1	1	1	1	0	0%	0%	5%	1%
Yaal_Hxk1p	0	0	0	0	0	0	0	0	1	0	0%	5%	1%
Yaph_Hxk1p1	0	0	0	0	0	0	0	0	1	0	0	5%	1%
Yaya_Hxk1p	28	28	28	28	28	28	28	28	29	28	28	0	6%
Cahi_Hxk1p	7	7	7	7	7	7	7	7	8	7	7	35	0

Table S2. Primers used in this study.

**Primers for gene overexpression**	
ScHXK2-BamHI-Fwd	GCGCGGATCCATGGTTCATTTAGGTCCAAAAAAACC
ScHXK2-AvrII-Rev	GCGCCCTAGGTTAAGCACCGATGATACCAACG
YayaHXK1-BglII-Fwd	GCGCAGATCTATGGTTCATCTTGGTCCCCGAAAAC
YayaHXK1-AvrII-Rev	GCGCCCTAGGCTAAATATCATACTTGACACCGGGCTTG
YlHXK1-CDS-BglII-Fwd	GAGAAGATCTATGGTTCATCTTGGTCCCCGAAAACCC
YlHXK1-S15A-BglII-Fwd	GCGCAGATCTATGGTTCATCTTGGTCCCCGAAAACCCCCGGCCCGAAAGG
YlHXK1-CDS-BglII-Δ7-15-Fwd	GAGAAGATCTATGGTTCATCTTGGTCCCCGAGCAG
YlHXK1-AvrII-Rev	GCGCCCTAGGCTAAATATCGTACTTGACACCGGGCTTG
YlHXK1-Δloop-Rev	GTGAAACCGAGAGGAATCTTGTGATCAAGAGCCTCAGGATAATTGTC
YlHXK1-Δloop-Fwd	AATTATCCTGAGGCTCTTGATCACAAGATTCCTCTCGGTTTCAC
YlHXK1-Δ152-182-Fwd	GTGAAACCGAGAGGAATCTTGTGATCAAGAGCCTCAGGATAATTGTC
YlHXK1-Δ152-182-Rev	GAAGCCAGGAGAACACGAAATATCTCGTCCTCGCTCATGGGC
YlHXK1-D386E-Rev	GAAAGAGGTCTCGAAGAAATACGAC
YlHXK1-D386E-Fwd	GTCGTATTTCTTCGAGACCTCTTTC
YlHXK1-E500G-Fwd	CAAAATCGTTCCCGCTGGAG
YlHXK1-E500G-Rev	CTCCAGCGGGAACGATTTTG
JMP62-pTEF-START	GGGTATAAAAGACCACCGTCC
JMP62-61STOP	GTAGATAGTTGAGGTAGAAGTTG
**Primers for gene disruption**	
P1-GLK1	ATAAGAATGCGGCCGCAGTCCGTGAGTTGTGGGGTC
P2-GLK1	CGATTACCCTGTTATCCCTACCGACAAAAAGAAGATACCCCCGCG
T1-GLK1	ATAAGAATGCGGCCGCGAGTGGCAGCATGTAGCTACTTG
T2-GLK1	ATAGTTTAGCGGCCGCCCGAATGTTTCTTACGCATGTCAC
P1-HXK1	ATAAGAATGCGGCCGCGGAGAGCATGGAGCAGAAAC
P2-HXK1	CGATTACCCTGTTATCCCTACCTGTTGCGGTAGAGAAATGCAG
T1-HXK1	GGTAGGGATAACAGGGTAATCGGTGGCACGTGTAGTTACCG
T2-HXK1	ATAGTTTAGCGGCCGCCTCGTTTCAATGGCTCCTCTTC
LAM001	CAGGAGTTATCCGAAGCGA

YlGLK1-ΔVer	TGTTGTGTTCGGCTCCAC
YlHXK1-ΔVer	CACATGACCTGATTCCCCAC
**qPCR primers**	
qACT1-FWD	AAGCGAGGTATCCTGACCCT
qACT1-REV	TTGGACTTGGGGTTGATGGG
qHXK1-FWD	TCTCCCAGCTTGAAACCATC
qHXK1-REV	CTTGACAACTCGCAGGTTGG
qTPS1-FWD	GTTTGGTACTGTCGAGTTTA
qTPS1-REV	AGTTTGACAAGCAACATACT
qLIP2-FWD	ACAATCAGATCGGCCCCAAG
qLIP2-REV	TTACCGACAATGGGCTGACC
qLIP8-FWD	AACGGTCTGGAAATCACCCC
qLIP8-REV	GTTTTCGCCTCCAGCACAAG
qLIP9-FWD	GCTTCGTTTGTTGGCTTGGT
qLIP9-REV	ACACAGTAGGAGACGGAGCA
qLIP13-FWD	CCATCTCGGAAGCCACCTAC
qLIP13-REV	CAAGCCCTTCAAACTGCGTC
qLIP17-FWD	CATCAACCCCGAAAGACGGA
qLIP17-REV	GGTCCAGCGCAGGAATAGAC
qYHT1-FWD	TCCAACCCCTTCCTCATCCA
qYHT1-REV	CAACAGCCGCCACGATAAAC
qYHT3-FWD	CTTCATGCCAGAGTCCCCTC
qYHT3-REV	GTGGCCTTACCAGCACTCTT
qYHT4-FWD	GCCGTGTCATCTCCGGTATT
qYHT4-REV	CAGGGCCCTTGATGTACGAG
qYALI0D09801g-FWD	GGCTCTTTTGGATACGCCCT
qYALI0D09801g-REV	GTATCGGCCCTTCTCGTGTT
qGUT1-FWD	CACCTACTTTGCCGGAGTCA
qGUT1-REV	GTTGGAGGTGTCGGTGATGT
qACS2-FWD	CGATACTGTGGCCGTCTACC
qACS2-REV	GGTGACGACAACCTTGGACT
qALK1-FWD	AGGAGACCAAGGACCCCATT
qALK1-REV	ACAGATCCTCACCGTCTCCA

File_S1

>YlHxk1p
MVHLGPRKPPSRKGSMADVPRDLLEQISQLETIFTVSPEKLRQITDHFVSELAKGLTKEG
GDIPMNPTWILGWPTGKESGCYLALDMGGTNLRVVKVTLDGDRGFDVMQSKYHMPPNIKV
GKQEELWEYIAECLGKFLADNYPEALDAHERGRDVDRTAAQSFTRDKSPPPHNQHISCSP
GFDIHKIPLGFTFSYPCSQPAVNRGVLQRWTKGFDIEGVEGEDVVPMLEAALERKNIPIS
ITALINDTTGTMVASNYHDPQIKLGNIFGTGVNAAYYEKVKDIPKLKGLIPDSIDPETPM
AVNCEYGAFDNEHKVLPRTKWDIIIDEESPRPGQQTFEKMSAGYYLGELLRLVLLDLYKD
GFVFENQGKNGQELGNGNINKSYFFDTSFLSLIEEDPWENLTDVEILFKEKLGINTTEPE
RKLIRRLAELIGTRSARISACGVAAICKKAGYKEAHAGADGSVFNKYPGFKERGAQALNE
IFEWNLPNPKDHPIKIVPAEDGSGVGAALCAALTIKRVKQGLPVGVKPGVKYDI
>Yake_Hxk1p
MVHLGPRKPPSRKGSMADVPRDLLEQISQLETIFTVSPEKLRQITDHFVSELAKGLTKEG
GDIPMNPTWILGWPTGKESGCYLALDMGGTNLRVVKVTLDGDRGFDVMQSKYHMPPNIKV
GKQEELWEYIAECLGKFLADNYPEALDAHERGRDVDRTAAQSFTRDKSPPPHNQHISCSP
GFDIHKIPLGFTFSYPCSQPAVNRGVLQRWTKGFDIEGVEGEDVVPMLEAALERKNIPIS
ITALINDTTGTMVASNYHDPQIKLGNIFGTGVNAAYYEKVKDIPKLKGLIPDSIDPETPM
AVNCEYGAFDNEHKVLPRTKWDIIIDEESPRPGQQTFEKMSAGYYLGELLRLVLLDLYKD
GFMFEHQGKNGQELGNGNINKSYFFDTSFLSLIEEDPWENLTDVEILFKEKLGIETTEPE
RKLIRRLAELIGTRSARISACGVAAICKKAGYKEAHAGADGSVFNKYPGFKERGAQALNE
IFEWNLPNPKDHPIKIVPAEDGSGVGAALCAALTIKRVKQGLPVGVKPGVKYDI
>Yaos_Hxk1p
MVHLGPRKPPSRKGSMADVPRDLLEQISQLETIFTVSPEKLRQITDHFVSELAKGLTKEG
GDIPMNPTWILGWPTGKESGCYLALDMGGTNLRVVKVTLDGDRGFDVMQSKYHMPPNIKV
GKQEELWEYIAECLGKFLADNYPEALEDHERGRDVDRTAAQSFTRDKSPPPHNQHISCSP
GFDIHKIPLGFTFSYPCSQPAVNRGVLQRWTKGFDIEGVEGEDVVPMLEAALERKKIPIS
ITALINDTTGTMVASNYHDPQIKLGNIFGTGVNAAYYEKVKDIPKLQGLIPDSIDPETPM
AVNCEYGAFDNEHKVLPRTKWDVIIDEESPRPGQQTFEKMSAGYYLGELLRLVLLDLYKD
GFVFEHQGKNGQELGNGNINKSYFFDTSFLSLIEEDPWENLTDVEILFKEKLGINTTEPE
RKLIRRLAELIGTRSARISACGVAAICKKAGYKEAHAGADGSVFNKYPGFKERGAQALNE
IFEWNLPNPKDHPIKIVPAEDGSGVGAALCAALTIKRVKQGLPVGVKPGVKYDI
>Yapo_Hxk1p
MVHLGPRKPPSRKGSMADVPRDLLEQISQLETIFTVSPEKLRQITDHFVSELARGLTKEG
GDIPMNPTWILDWPTGKESGCYLALDMGGTNLRVVKVTLDGDRGFDVMQSKYHMPPNIKV
GKQEELWEYIAECLGKFLADNYPEALEDHERGRDVDRTAAQSFTRDKSPPPHNQHISCSP
GFDIHKIPLGFTFSYPCSQPAVNRGVLQRWTKGFDIEGVEGEDVVPMLEAALERKNIPIS
ITALINDTTGTMVASNYHDPQIKLGNIFGTGVNAAYYEKVKDIPKLKGLIPDSIDPETPM
AVNCEYGAFDNEHKVLPRTKWDIIIDEESPRPGQQTFEKMSAGYYLGELLRLVLLDLYKD
GFVFEHQGKNGKELGNGNINKSYFFDTSFLSLIEEDPWENLTDVEILFKEKLGIDTTEPE
RKLIRRLAELIGTRSARISACGVAAICKKAGYKEAHAGADGSVFNKYPGFKERGAQALNE
IFEWNLPNPKDHPIKIVPAEDGSGVGAALCAALTIKRVKQGLPVGVKPGVKYDI
>Yade_Hxk1p
MVHLGPRKPPSRKGSMADVPRDLLEQISQLETIFTVSPEKLRQITDHFVDELAKGLTKEG
GDIPMNPTWILGWPSGKESGCYLALDMGGTNLRVVKVTLDGDRGFDVMQSKYHMPPNIKV
GKQEELWEYIAECLGKFLADNYPEALDDHERGRDVDRTAAQSFTRDKSPPPHNQHISCSP
GFDIHKIPLGFTFSYPCSQPAVNRGVLQRWTKGFDIEGVEGEDVVPMLEAALERKNIPIS
ITALINDTTGTMVASNYHDPQIKLGNIFGTGVNAAYYEKVKDIPKLKGLIPESIDPETPM
AVNCEYGAFDNEHKVLPRTQWDITIDEESPRPGQQTFEKMSAGYYLGELLRLVLLDLYKD
GFMFETQGKNGQELGNGNINKSYFFDTSFLSLIEEDPWENLTDVEILFKDKLGINTTEPE
RKLIRRLAELIGTRSARISACGVAAICKKAGYKEAHAGADGSVFNKYPGFKERGAQALNE

IFEWNLPNPKDHPIKIVPAEDGSGVGAALCAALTIKRIKQGLPVGVKPGVKYDI
>Yadi_Hxk1p
MVHLGPRKPPSRKGSMADVPRDLLEQISQLETIFTVSPEKLRQITDHFVDELAKGLTKEG
GDIPMNPTWILGWPTGKESGCYLALDMGGTNLRVVKVTLDGDRGFDVMQSKYHMPPNIKV
GKQEELWEYIAECLGKFLADNYPEALDDHERGRDVDRTAAQSFTRDKSPAPHNQHISCSP
GFDVHKIPLGFTFSYPCSQPAVNRGVLQRWTKGFDIEGVEGEDVVPMLEAALERKNIPIS
ITALINDTTGTMVASNYHDPQIKLGNIFGTGVNAAYYEKVKDIPKLKGLIPESIDPETPM
AVNCEYGAFDNEHKVLPRTQWDITIDEESPRPGQQTFEKMSAGYYLGELLRLVLLDLYKD
GFMFETQGKNGQELGNGNINKSYFFDTSFLSLIEEDPWENLTDVEILFKDKLGINTTEPE
RKLIRRLAELIGTRSARISACGVAAICKKAGYKEAHAGADGSVFNKYPGFKERGAQALNE
IFEWNLPNPKDHPIKIVPAEDGSGVGAALCAALTIKRIKQGLPVGVKPGVKYDI
>Yaga_Hxk1p
MVHLGPRKPPSRKGSMADVPRDLLEQISQLETIFTVSPEKLRQITDHFVSELAKGLTKEG
GDIPMNPTWILGWPTGKESGCYLALDMGGTNLRVVKVTLDGDRGFDVMQSKYHMPPNIKV
GKQEELWEYIAECLGKFLADNYPEALEDHERGRDVDRTAAQSFTRDKSPAPHNQHISCSP
GFDVHKIPLGFTFSYPCSQPAVNRGVLQRWTKGFDIEGVEGEDVVPMLEAALERKKIPIS
ITALINDTTGTMVASNYHDPQIKLGNIFGTGVNAAYYEKVKDIPKLKGLIPESIDPETPM
AVNCEYGAFDNEHKVLPRTKWDIIIDEESPRPGQQTFEKMSAGYYLGELLRLVLLDLYKD
GFMFENQGKNGQDLGNGNINKSYFFDTSFLSLIEEDPWENLTDVEILFKDKLGITTTEPE
RKLIRRLAELIGTRSARISACGVAAICKKAGYKEAHAGADGSVFNKYPGFKERGAQALNE
IFEWNLPNPKDHPIKIVPAEDGSGVGAALCAALTIKRIKQGLPVGVKPGVKYDI
>Yabu_Hxk1p
MVHLGPRKPPSRKGSMADVPRDLLEQISQLETIFTVSPEKLRQITDHFVAELAKGLTKEG
GDIPMNPTWILDWPTGKESGCYLALDMGGTNLRVVKVTLDGERGFDVMQSKYHMPPNIKV
GKQEELWEYIAECLGKFLADNYPEALDDHERGRDVDRTAAQSFTRDKSPPPHNQHISCSP
GFDIHKIPLGFTFSYPCSQPAVNRGVLQRWTKGFDIEGVEGEDVVPMLEAALERKNIPIS
ITALINDTTGTMVASNYHDPQIKLGNIFGTGVNAAYYEKVKDIPKLKGLIPDSIDPETPM
AVNCEYGAFDNEHKVLPRTQWDVIIDEDSPRPGQQTFEKMSAGYYLGELLRLVLLDLYKD
GFVFEHQGKNGTELGNGNINKSYFFDTSFLSLIEEDPWENLTDVEILFKEKLGIHTTEPE
RKLIRRLAELIGTRSARISACGVAAICKKAGYKEAHAGADGSVFNKYPGFKERGAQALNE
IFEWNLPNPKDHPIKIVPAEDGSGVGAALCAALTIKRVKLGLPVGVKPGVKYDI
>Yaho_Hxk1p
MVHLGPRKPPSRKGSMADVPRDLLEQISQLETIFTVSPEKLRQITDHFVSELAKGLTKEG
GDIPMNPTWILDWPTGKESGCYLALDMGGTNLRVVKVTLDGDRGFDVMQSKYHMPPNIKV
GKQEELWEYIAECLGKFLADNYPEALEQHERGRDVDRTAAQTFTRDKSPAPHNLHISCSP
GFDIHKIPLGFTFSYPCSQPAVNRGVLQRWTKGFDIEGVEGEDVVPMLEAALERKNIPIS
ITALINDTTGTMVASNYHDPQIKLGNIFGTGVNAAYYEKVKDIPKLKGLIPDSIDPETPM
AVNCEYGAFDNEHKVLPRTKWDIIIDEESPRPGQQTFEKMSAGYYLGELLRLVLLDLYKD
GFVFENQGKSGELGNGNINKSYFFDTSFLSLIEEDPWENLTDVEILFKDKLGIDTTEPER
KLIRRLAELIGTRSARISACGVAAICKKAGYEKAHAGADGSVFNKYPGFKERGAQALNEI
YEWNLPNPKDHPIKIVPAEDGSGVGAALCAALTIKRVKLGLPVGVKPGVKYDI
>Yaal_Hxk1p
MVHLGPRKPPSRKGSMADVPRDLLEQISQLETIFTVSADKLRQITDHFVKELAKGLTKEG
GDIPMNPTWILDWPTGKETGCYLALDMGGTNLRVVKVTLDGDRGFDVMQSKYHMPPNIKV
GKQEELWEYIAECLGKFLADNYPEALAQHERGRDVDRTAAQGFTRDKSPAPHNLHISCSP
GVDVHKIPLGFTFSYPCSQPAVNRGVLQRWTKGFDIEGVEGEDVVPMLEAALERKNIPIS
ITALINDTTGTMVASNYHDPQIKLGNIFGTGVNAAYYEKVKDIPKLAGLIPDSIDPETPM
AVNCEYGAFDNEHKVLPRTKWDVIIDEESPRPGQQTFEKMSAGYYLGELLRLVLLDLYND
GFMFERQGKNGETLGNGNINKSYFFDTSFLSLIEEDPWENLTDVEILFKDKLGINTTEPE
RKLIRRLAELIGTRSARISACGVAAICKKAGYKEAHAGADGSVFNKYPGFKERGAQALNE
IFEWNLPNPKDHPIKIVPAEDGSGVGAALCAALTIKRVKAGLPVGVKPGVKYDI

>Yaph_Hxk1p
MVHLGPKKPPSRKGSMADVPRDLLEQVSQLESLFIVSPEKLRQITDHFVSELARGLTKEG
GDIPMNPTWILDWPTGDETGCFLALDMGGTNLRVVKVTLDGERGFDVMQSKYHMPPNIKV
GKQEELFDYIAECLGKFLADNYPEALEQHERGRDVDRTASQEFTRDKSPAPHNHHISCAP
GVDIHKIPLGFTFSYPCSQPAVNRGVLQRWTKGFDIEGVEGEDVVPLLEAALDKKNIPIT
ITALINDTTGTLVASNYHDPQIKLGNIFGTGVNAAYYEKVKDIPKLKGLIPESIDPETPM
AVNCEYGAFDNEHKVLPRTKWDVIIDDESPRPGQQTFEKMSAGYYLGELLRLVLLDLYAD
GFIFETQGKNGQELGNGNIKKSYFFDTSFLSLIEEDPWENLTDVEILFKDKLSIDTTEPE
RKLIRRLAELIGTRAARISACGVAAICKKAGYKEAHAGADGSVFNKYPGFKERGAQALNE
IFEWNLPNPKDHPIKIVAAEDGSGVGAALCAALTNKRVTQGLPVGSKPGVKYDI
>Yaya_Hxk1p
MVHLGPRKPPSRKGSMADVPRDLLEQISQLETIFTVSPEKLRQITDRFVSELARGLTKEG
GDIPMNPTWILDWPTGKETGCYLALDMGGTNLRVVKVTLDGERGFDVMQSKYHMPPSIKV
GKQEELWEYIAECLGKFLAQNYPESLDESSRAESDINKIPLGFTFSYPCSQPAVNRGVLQ
RWTKGFDIDGVEGEDVVPMLVAALERKNIPISVTALINDTTGTMVASNYHDPQIKLGNIF
GTGVNAAYYEKVKDIPKLKGLIPESIDPETPMAVNCEYGAFDNEHKVLPRTKWDIIIDEE
SPRPGQQTFEKMSAGYYLGELLRLVLLDLYKDGFIFEHQGKNGEELGNGNINKSYFFDTS
FLSLIEEDPWENLTDVEILFKEKLGISTTEPERKLIRRLAELIGTRAARISACGVAAICK
KAGYNEAHAGADGSVFNKYPGFKERGAQALNEIYEWNLPNPKDHPIKIVPAEDGSGVGAA
LCAALTIKRVKQGLPVGAKPGVKYDI
>Cahi_Hxk1p
MVHLGPRKPPSRKGSMADVPRDLLDEVKKLEEIFIVSPELLKKITDHFVDELTKGLSEEG
GSIPMNPTWIIDWPTGKETGCYLALDMGGTNLRVVKVTLDGNRGFDIMQSKYHMPSDIKC
SSKEKLWDYIADCLGKFLEDNHPHVLKDRGRQSFDEANGDAAEHAFCRDKSPAPHNQHIS
CADNTGGDDDKLPLGFTFSYPCSQPAVNQGILQRWTKGFDIDGVEGEDVVPMLEQALDKK
NIPIQVTALINDTTGTLVASNYADVRIKLGNIFGTGVNAAYYEKVKNIPKLAKYLPEGLD
PETPMAVNCEYGAFDNEGVVLPRTKWDTAIDDASPRPGQQRFEKMTAGYYLGEILRRVLL
DLHEDGHIFVKETKGKIGNAKIHKEYVLDASFLSRIEEDPWENLSDVEELFLELLGISTN
PFERKLIRRLAELIGTRAARISACGVAAICKKAGYSEAHAGADGSVFNKYPGFKERGAQA
LREIFDWEIEDPKDYPIKIVPAEDGSGVGAALCAAITNKRIRKGLPVGAPKGMDYTI
